# Making Waves in the Brain: What Are Oscillations, and Why Modulating Them Makes Sense for Brain Injury

**DOI:** 10.3389/fnsys.2016.00030

**Published:** 2016-04-07

**Authors:** Aleksandr Pevzner, Ali Izadi, Darrin J. Lee, Kiarash Shahlaie, Gene G. Gurkoff

**Affiliations:** ^1^Department of Neurological Surgery, University of California-DavisSacramento, CA, USA; ^2^Center for Neuroscience, University of California-DavisSacramento, CA, USA

**Keywords:** traumatic brain injury, electrical neuromodulation, deep brain stimulation, oscillations, hippocampus, theta

## Abstract

Traumatic brain injury (TBI) can result in persistent cognitive, behavioral and emotional deficits. However, the vast majority of patients are not chronically hospitalized; rather they have to manage their disabilities once they are discharged to home. Promoting recovery to pre-injury level is important from a patient care as well as a societal perspective. Electrical neuromodulation is one approach that has shown promise in alleviating symptoms associated with neurological disorders such as in Parkinson’s disease (PD) and epilepsy. Consistent with this perspective, both animal and clinical studies have revealed that TBI alters physiological oscillatory rhythms. More recently several studies demonstrated that low frequency stimulation improves cognitive outcome in models of TBI. Specifically, stimulation of the septohippocampal circuit in the theta frequency entrained oscillations and improved spatial learning following TBI. In order to evaluate the potential of electrical deep brain stimulation for clinical translation we review the basic neurophysiology of oscillations, their role in cognition and how they are changed post-TBI. Furthermore, we highlight several factors for future pre-clinical and clinical studies to consider, with the hope that it will promote a hypothesis driven approach to subsequent experimental designs and ultimately successful translation to improve outcome in patients with TBI.

## Introduction

There are an estimated 3.8 million new traumatic brain injury (TBI) cases annually, and well over 5.3 million patients report chronic TBI-related deficits (Langlois et al., 2006; DeKosky et al., 2010). Ultimately, an estimated $221 billion (combined acute and chronic care) is spent to treat TBI annually (Coronado et al., 2012). Critically, however, only 7% ($14.6 billion) of the estimated $221 billion is spent on direct medical costs (Coronado et al., 2012) and therefore, the vast majority of the financial burden is related to the long-term care of patients with chronic disabilities. In addition to the financial cost, there is a significant and well-documented emotional toll of caring for chronic TBI patients both on caregivers and society at large (Roozenbeek et al., 2013). Therefore, there is a critical need to develop innovative strategies to specifically address and improve the quality of life for patients with chronic disability following TBI. In the following review, we propose that oscillations observed in the electroencephalogram (EEG) play a key role in cognitive function and that a TBI-induced change in oscillations can result in impaired behavioral function. Finally, we discuss the potential for electrical neurostimulation to improve chronic behavioral outcome in TBI patients.

## A Review of the Immediate Effects of TBI

The application of mechanical force on the brain initiates a complex series of interacting (sometimes non-monotonic) biochemical cascades, which, along with the initial impact, characterize TBI pathophysiology. Disruption of the cell membrane can lead to an ionic disturbance of Na^+^, K^+^, Ca^2+^, Mg^2+^, and Zn^2+^ (Vink et al., 1988; Katayama et al., 1990; Soares et al., 1992; Nilsson et al., 1993; Smith et al., 1993). The high concentration of K^+^ and Ca^2+^ in the extracellular space triggers release of neurotransmitters (e.g., glutamate), which can further exacerbate the ionic disturbance creating a vicious cycle (Faden et al., 1989; Katayama et al., 1990; Nilsson et al., 1990; Lyeth et al., 1993; Reeves et al., 1997; Shin and Dixon, 2015). This wave of depolarization can lead to excitotoxic cell death beyond what is observed in the injury core and surrounding penumbra (Sullivan et al., 1976; Dixon et al., 1987; Lowenstein et al., 1992; Hicks et al., 1993; Yamaguchi et al., 1996; Leonard et al., 1997; Yakovlev et al., 1997; Floyd et al., 2002; Witgen et al., 2005; Fedor et al., 2010). Moreover, there is considerable evidence that the pathophysiological release of neurotransmitters can alter the function of glutamatergic (Faden et al., 1989; Miller et al., 1990; Smith et al., 1993; Schwarzbach et al., 2006), cholinergic (Robinson et al., 1990; Yamamoto et al., 1993; Jiang et al., 1994; Lyeth et al., 1994; Delahunty et al., 1995; Shin and Dixon, 2015), GABAergic (Reeves et al., 1997; Witgen et al., 2005; Bonislawski et al., 2007; Gupta et al., 2012), and dopaminergic receptor systems (Donnemiller et al., 2000; Massucci et al., 2004; Shin et al., 2011), resulting in potential long-term cellular and circuit dysfunction independent of cell death.

One specific change related to excessive activation of the nervous system following TBI is the accumulation of intracellular calcium and the subsequent activation of calcium dependent catalytic enzymes such as calpain (Kampfl et al., 1997; Khorchid and Ikura, 2002). While hyperactivation of calpains is commonly associated with apoptosis (Patel et al., 1996), calpains also advance cytoskeletal and plasma membrane breakdown as well as disruption of Na^+^ channel function (Hicks et al., 1995; Folkerts et al., 1998; Saatman et al., 1998; Johnson et al., 2013). Changes in the cytoskeleton and membrane can trigger further ionic imbalance and specifically lead to high intra-axonal Ca^2+^ levels, which further challenge the already damaged axons resulting from the primary injury (Graham et al., 2000; Kita et al., 2000; Baker et al., 2002; Johnson et al., 2013; Li et al., 2014). Not surprisingly, for a prolonged period after the initial injury, neurons in the corrupted neural network can have impaired neurophysiological responses (Reeves et al., 1997; Golarai et al., 2001; Santhakumar et al., 2001; Kao et al., 2004; Goforth et al., 2011) including long-term potentiation (LTP; Miyazaki et al., 1992; Reeves et al., 1995; D’Ambrosio et al., 1998; Sick et al., 1998; Sanders et al., 2000; Schwarzbach et al., 2006; Li et al., 2014). In addition, TBI results in deficits impacting certain forms of behavioral plasticity (Ip et al., 2002; Griesbach et al., 2004) and formation of long term memories (Rimel et al., 1981; Leininger et al., 1990; Fedor et al., 2010; Gurkoff et al., 2013; Zhang et al., 2015). Disruption to cognition and plasticity following TBI is of particular relevance to our research interests and will be the focus of this review. Specific emphasis will be placed on how oscillatory activity contributes to information processing and how modifying injury- perturbed EEG could be relevant to reversing deficits in the clinical population. To this end we will first elaborate on what local field oscillations are and how they are generated in the brain.

## Defining an Oscillation

There is both growing evidence and excitement that neuromodulation, and specifically invasive electrical neurostimulation, can be used to improve function in patients with neurological disorders (Lozano and Lipsman, 2013; Suthana and Fried, 2014; Tekriwal and Baltuch, 2015). In the case of TBI it is clear that functional consequences can be severe and persist for many years after the insult (Jennett et al., 1981; Whiteneck et al., 2004; DeKosky et al., 2010; Ponsford et al., 2014). And at least some of these cognitive and behavioral deficits could be mitigated with neurostimulation (Buzsáki and Watson, 2012; Lozano and Lipsman, 2013; Shin et al., 2014). Although the precise mechanism is still being delineated, it is hypothesized that driving specific neural circuits can entrain physiological circuit activity ultimately improving behavioral outcomes. Stemming from this, based on our recent findings we hypothesize that TBI-induced alterations in neural connectivity result in altered oscillations, as observed in the EEG. Further, we hypothesize that stimulating the injured nervous system to restore or substitute these oscillations will improve outcome. However, essential to the implementation and assessment of any intervention is the knowledge of the underlying mechanisms involved. Therefore, the next sections will summarize the basic neurophysiology associated with brain oscillations observed in the EEG and how these oscillations contribute to neural function. Critically this overview will introduce concepts from the perspective of developing research strategies to determine whether electrical neurostimulation can be used to improve cognitive outcome in TBI patients.

EEG is the measurement of change in the extracellular field potential recorded from the scalp that is generated by the sum of ionic movements across synapses, dendrites, soma, axons and the electroconductive cerebral spinal fluid. Similar activity measured from intracranial electrodes is commonly referred to as intracranial EEG (iEEG) or electrocorticography (ECoG). For simplicity, we will refer to all recordings, scalp and intracranial, as EEG for the remainder of this manuscript. The average of ionic movements within the immediate surrounding volume of an implanted electrode is referred to as the *local* field potential (LFP). There are many cellular actions that sum together to contribute to the total change in the ionic balance measured by a depth electrode, such as synaptic activity, Ca^2+^ fluctuations, intrinsic currents and resonances, spike after-hyperpolarization, gap junctions and glial interaction (Berridge and Rapp, 1979; Buzsáki et al., 2012). The magnitude of the electric field detected in the EEG is related to the alignment of the electrode relative to the processes of cells in any given region (Buzsáki et al., 1986; Montgomery et al., 2009). Specifically, an electrode placed parallel to the dipole created by ionic movements will result in the highest amplitude recording (Kringelbach et al., 2007).

A synchronized and reoccurring change in ionic movements results in an oscillation that can be observed in the EEG. Oscillations may arise due to a variety of mechanisms, such alternating excitation-inhibition (or excitation-excitation or inhibition-inhibition) of neurons, pacemaker cells, resonance or subthreshold membrane oscillation (James et al., 1977; Buzsáki et al., 1983; McCormick and Bal, 1997; Marshall et al., 2002; Klausberger et al., 2003; Wang, 2010). There are multiple discrete oscillatory bands ranging from 0.05 to 500 Hz that have been operationally defined based on functional states of the brain (Klausberger et al., 2003; Penttonen and Buzsáki, 2003; Buzsáki and Watson, 2012). While the general structure of many oscillations is similar (e.g., alternating excitation-inhibition, pacemaker cell), granularly each rhythm is quite distinct from one other. How, when, and where an oscillation is generated defines its operation and contribution to information processing, and in the case of a brain injury, the pathophysiology of a disorder. Therefore, in order to understand how TBI might affect the generation or maintenance of oscillations, and how to develop and assess potential strategies to restore oscillations, it is critical to consider how mechanistically an oscillation is generated.

## Understanding How Oscillations are Generated

One of the earliest and most studied examples of oscillations observed in the EEG is from studies of sleep progression. For example, a defining characteristic of early non-REM (NREM) sleep is the presence of spindle waves, which are 1–3 s bursts of activity in the 7–14 Hz range every 3–10 s (Brown et al., 2012). To describe spindle generation it is important to consider both which brain regions as well as which specific cellular mechanisms are responsible for generating rhythmicity. Spindles arise due to the thalamic reticular nucleus (TRN) hyperpolarizing thalamocortical neurons with a rhythmic burst of inhibitory synaptic potentials (IPSPs; Avanzini et al., 1989; Bal et al., 1995a,b). This hyperpolarization leads to the activation of low-threshold T-type Ca^2+^ channels (I_T_), which even at low, negative membrane potentials can generate an action potential. Subsequently thalamocortical neurons generate a burst of excitatory synaptic potentials (EPSPs) that activate the TRN as well as corticothalamic neurons giving rise to a spindle (Crunelli et al., 1989; Bal et al., 1995a,b). Convergence of excitatory input onto TRN activates low-threshold Ca^2+^ channels, which send prolonged IPSPs back to thalamocortical neurons starting the oscillatory cycle anew (Steriade and Deschenes, 1984; Avanzini et al., 1989; McCormick and Bal, 1997). Thus, the time to go through one full cycle prescribes the observed frequency of a spindle (Bal et al., 1995a).

Thalamocortical bursting activity gives rise to another dominant NREM sleep oscillation in the delta frequency band (0.5–4 Hz; McCormick and Bal, 1997; Brown et al., 2012). Unlike the spindle waves, delta oscillations are generated in a single cell by the interplay between ionic currents (Steriade et al., 1993b). Low-threshold Ca^2+^ bursting in thalamocortical neurons is followed by a hyperpolarizing overshoot. This de-inactivates I_T_ and opens the hyperpolarization-activated cation channel causing an h-current (I_h_). I_h_ slowly depolarizes the cell towards the threshold for a Ca^2+^ spike by activating I_T_. Depolarization past −65 mV and subsequently −35 mV inactivates I_T_ and deactivates I_h_, respectively, and leads to an action potential (Crunelli et al., 1989; McCormick and Bal, 1997). Repolarization overshoots start the cycle again. However, it should be noted that other mechanisms have been proposed to account for the thalamocortical delta oscillation (Ball et al., 1977; Steriade et al., 1993a).

Specific to our understanding of oscillations during sleep it is easy to imagine how the precise activity of a series of receptor systems and the related interaction of ionic currents would be sensitive to the large ionic imbalance that follows TBI (Vink et al., 1988; Katayama et al., 1990; Soares et al., 1992; Nilsson et al., 1993; Smith et al., 1993). Consistent with this assertion, TBI is associated with sleep disturbances (Mathias and Alvaro, 2012) and specifically a decrease in delta power during NREM sleep for at least 12 weeks post injury (Parsons et al., 1997). Therefore, when considering how TBI alters oscillations and the potential for neurostimulation one has to determine not only which circuits and specific mechanisms are affected, but also when one needs to stimulate.

**Figure 1:**
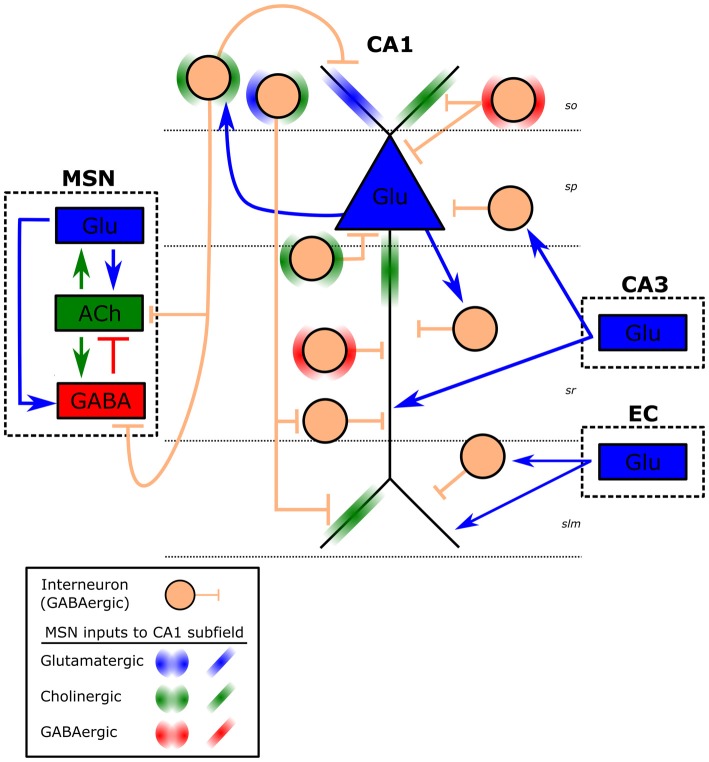
**Schematic of CA1 theta generators.** Illustrated is a CA1 pyramidal cell (blue triangle) and hippocampal GABAergic interneurons (peach circle) within each CA1 layer. Approximate CA1 layers are indicated by dashed horizontal lines (so, stratum oriens; sp, stratum pyramidale; sr, stratum radiatum; slm, stratum lacunosum-moleculare). Interneurons within each layer represent a subclass of interneurons (e.g., O-LM, PV basket, axo-axonic) for that layer, which receive different inputs and have distinct projections (e.g., back projecting). Dashed boxes represent CA1 inputs from medial septal nucleus (MSN), entorhinal cortex (EC) and the CA3 subfield. Arrows represent excitatory (blue-Glu: glutamate, green: ACh- acetylcholine), whereas short vertical lines are inhibitory (red and peach: GABA) connections. Projections from the MSN are left out for clarity and are represented by halos (circular on interneurons, linear on CA1 pyramidal cell).

Our primary interest related to TBI and EEG is how injury may alter hippocampal oscillations and cognitive function. This interest is driven by a rich history in TBI-induced spatial learning deficits, deficits that we now know are concurrent with altered hippocampal oscillations (Fedor et al., 2010; Lee et al., 2013, 2015). Unlike the previously described oscillations, hippocampal theta (3–12 Hz), and specifically in CA1 is generated and maintained by the interaction of multiple rhythm generators as well as intrinsic membrane properties of hippocampal neurons that contribute to the detected rhythmic slow wave (Green and Arduini, 1954; Vanderwolf, 1969; Buzsáki et al., 1986; Kirk, 1998; Kocsis et al., 1999; Mormann et al., 2008; Montgomery et al., 2009; Colgin, 2013; Watrous et al., 2013). In the hippocampal CA1 subfield there are two well characterized dipoles of theta: the distal dendrites and soma (Figure 1). The first dipole, measured strongest near the hippocampal fissure, is attributed to layer 3 entorhinal cortex (EC) and CA3 subfield rhythmic excitation of distal dendrites of CA1 (Bland, 1986; Alonso and García-Austt, 1987; Konopacki et al., 1987; Kamondi et al., 1998; Kocsis et al., 1999). This dendritic depolarization co-occurs with somatic hyperpolarization, which reflects inputs from the medial septum nucleus (MSN; Green and Arduini, 1954; Petsche et al., 1962; James et al., 1977; Bland, 1986; Vertes et al., 2004). The MSN consists of three types of neurons: GABAergic, cholinergic and glutamatergic. In fact, afferents from each of these neuronal subtypes play a role in the generation of the second dipole. Specifically, the interplay of phasic GABAergic inhibition, tonic cholinergic and glutamatergic excitation of hippocampal interneurons results in CA1 theta (Cole and Nicoll, 1984; Smythe et al., 1992; Tóth et al., 1997; Apartis et al., 1998; Wang, 2002; Hajszan et al., 2004; Colom et al., 2005; Vandecasteele et al., 2014; Fuhrmann et al., 2015). The septal GABAergic cells act as pacemakers of theta generation in CA1 pyramidal cells through disinhibiting hippocampal interneurons (Freund and Antal, 1988; Ylinen et al., 1995; Wang, 2002). In addition, MSN cholinergic and glutamatergic neurons directly modulate excitability in CA1 pyramidal cells, which in turn excite back projecting hippocamposeptal interneurons completing the septohippocampal loop (Figure 1; Gaykema et al., 1991; Tóth and Freund, 1992; Tóth et al., 1993; Manseau et al., 2008; Mattis et al., 2014; Sun et al., 2014). This interplay between septohippocampal interneurons has the added effect of disinhibiting and inhibiting the soma of CA1 pyramidal neurons at the theta frequency, which can be measured at or dorsal to the pyramidal layer. Interestingly, hippocampal interneurons are vulnerable to cell death after TBI (Tóth et al., 1997b; Almeida-Suhett et al., 2015; Huusko et al., 2015). In addition there is evidence that injury can alter function in these neurons (O’Dell et al., 2000; Ross and Soltesz, 2000; Mtchedlishvili et al., 2010; Gupta et al., 2012; Almeida-Suhett et al., 2015; Drexel et al., 2015). Any change in interneuronal number or function could contribute to changes seen in the theta band post injury. While it is well accepted that TBI can result in cell death and dysfunction in interneurons in general, in order to get a better understanding of the hippocampal pathophysiology it will be important for future studies to examine which specific classes of interneurons (Figure 1, e.g., O-LM, PV basket, axo-axonic) that contribute to CA1 theta generation are also affected by TBI (Klausberger et al., 2003).

These theta rhythm generators also work in concert with the intrinsic properties of hippocampal neurons. Specifically, there are intrinsic resonant and subthreshold membrane oscillating events which contribute to the hippocampal oscillations. For example, CA1 pyramidal cells have resonance (preferred frequency for maximal response) at theta frequency due to the interplay between voltage gated ionic currents. Depolarizations activate I_M_ (K^+^ current), which has the effect of hyperpolarizing the cell. Hyperpolarization activates I_h_ (mix Na^+^/K^+^ current), which brings the potential closer to spike threshold. With the addition of a persistent Na^+^ current (I_NAP_) these currents oppose each other resulting in a membrane resonance in the theta frequency (Pike et al., 2000; Hu et al., 2002). This CA1 rhythmicity is further amplified with subthreshold membrane oscillations via persistent Na^+^ and Ca^2+^ currents (Leung and Yim, 1991; García-Muñoz et al., 1993; Fransén et al., 2004). In a similar vein, both EC cells projecting to the hippocampus (Alonso and Llinas, 1989; Alonso and Klink, 1993; Klink and Alonso, 1993; Dickson et al., 2000; Quilichini et al., 2010) and hippocampal inhibitory interneurons (Maccaferri and McBain, 1996; Chapman and Lacaille, 1999; Pike et al., 2000) have a natural resonance and subthreshold membrane oscillation in the theta frequency range due to a mix of voltage-sensitive Na^+^ and K^+^ currents. Furthermore, the MSN displays intrinsic bursting in the theta range (Vinogradova et al., 1980; Zhadina and Vinogradova, 1983). Thus, the magnitude of synaptically driven theta from the generators in the EC and septum is boosted by multiple intrinsic resonances from cells within the hippocampus as well as extrinsic to the hippocampal formation (Goutagny et al., 2009).

In summary, there are bands of oscillations starting as low as <0.1 Hz and ranging to as high as 600 Hz. Over the years we have operationally defined discrete ranges of oscillations (e.g., theta and gamma) based on specific cellular mechanisms as they relate to observed behavioral relationships. Research into individual oscillatory bands has revealed that mechanisms for the generation and maintenance of oscillations are complex and varied, with interactions of synaptic and intrinsic generators summing together to provide a single detected change in the LFP. However, it remains an open question to what extent TBI alters any, or perhaps all, of the specific mechanisms involved in the generation of individual oscillatory bands and ultimately the neural network underlying cognition.

## How Oscillations Interact and Contribute to Information Processing

**Figure 2:**
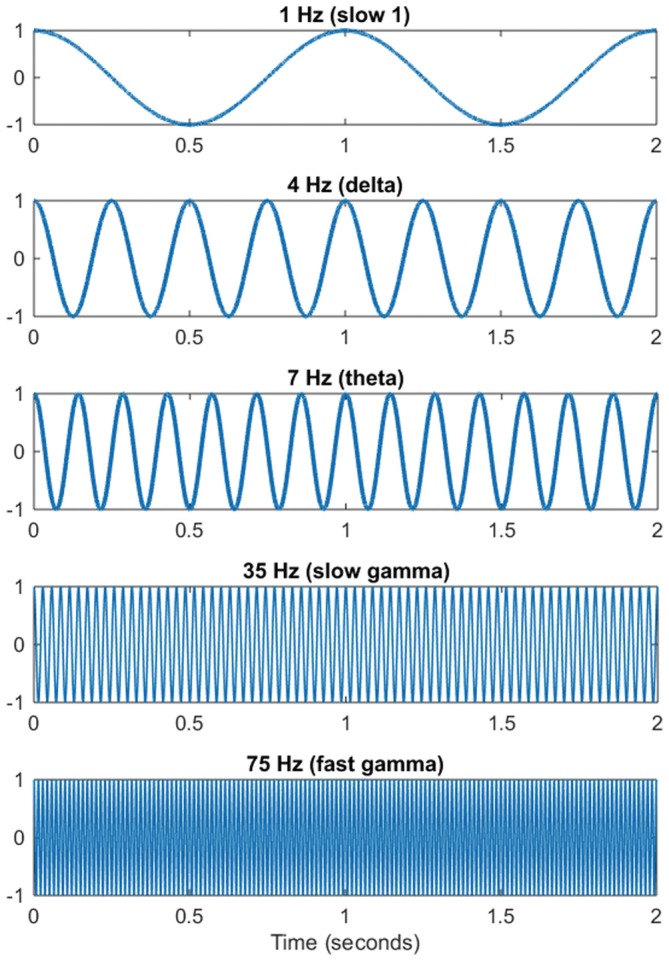
**Oscillations of varying frequency.** MATLAB generated sine curves to represent neuronal oscillations that make up the unfiltered electroencephalogram (EEG). Each panel represents a distinct frequency and in parentheses the corresponding oscillatory band name most often associated with it.

While each electrode yields a single LFP measure, that LFP is made up of several components. In fact, the combination of synaptic and intrinsic membrane events frequently leads to power in multiple oscillatory bands. Specifically, when one decomposes an individual LFP one can see that each oscillation occurs simultaneously (Figure 2). Figure 2 illustrates sinusoidal waves whose frequency corresponds to the slow, delta, theta and gamma oscillatory bands. A more exhaustive list of oscillatory frequencies was described by Penttonen and Buzsáki (2003). In general slow wave oscillations, and relevant to the current discussion those in the theta frequency range, are thought to synchronize distal regions of the brain promoting plasticity, while faster gamma oscillations are hypothesized to link and/or activate local neuronal ensembles (Bragin et al., 1995; Penttonen and Buzsáki, 2003; Buzsáki and Draguhn, 2004). There are several published reviews relating to a broader analysis of EEG and their role in plasticity and learning (Başar et al., 2001; Buzsáki, 2005; Lakatos et al., 2008; Knyazev, 2012; De Gennaro and Ferrara, 2003). However, based on the current level of understanding of these oscillations as they pertain to TBI is limited and therefore an in depth description of these findings is beyond the scope of this review. But, if we want to understand the extent of the effect of TBI on oscillations, it is important to not only consider one specific frequency band at a single electrode, for example hippocampal theta, but instead consider how multiple frequency bands are related at a single recording site (i.e., cross frequency coupling), and also how similar frequency bands are related between distal electrodes (i.e., phase coherence). Thus, in order to better understand the effects of brain injury on EEG it will be necessary to sample from multiple regions within a circuit as well as to investigate a range of frequency interactions in addition to a power analysis.

**Figure 3:**
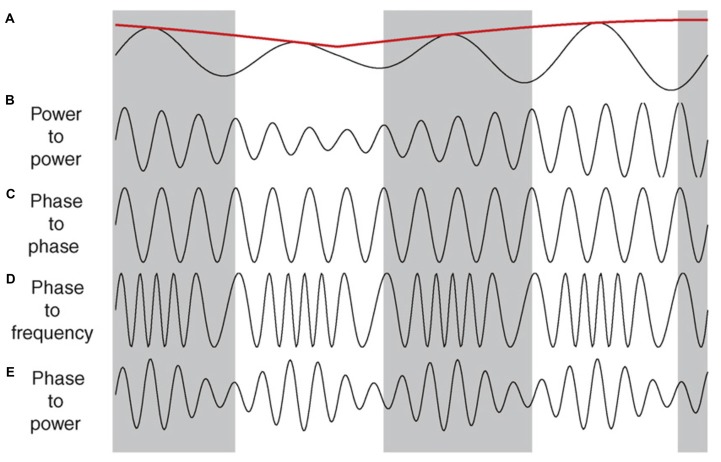
**Illustrations of cross-frequency coupling. (A)** A slow oscillation in the theta range, along with its power indicated by the red line. **(B–E)** Illustrate the different types of interplay that faster oscillations (e.g., gamma) can have with the slower trace in **(A)**. **(B)** Power–power: the power (amplitude) of the faster wave correlates with the power of the slower wave. **(C)** Phase–phase: a fixed number of faster cycles within each phase of the slower oscillation. In this case there are four cycles within each phase. **(D)** Phase–frequency: the number of cycles in the faster wave correlates with specific phase of the slower wave. **(E)** Phase–power: the power of the faster wave correlates with the specific phase of the slower wave, independent of the power of the slower wave. Reproduced with permission from Elsevier (Jensen and Colgin, 2007).

Interactions that take place across different frequencies at a single recording site are referred to as cross frequency coupling. The interplay between two frequencies could take place across several different domains (Figure 3). For example, cross-frequency power-power coupling (amplitude-amplitude) occurs when the power of the low frequency oscillation dictates the power of the high frequency oscillation; cross-frequency phase-phase coupling (n:m phase locking) refers to a fixed number of high frequency oscillations nested in each slower cycle; cross-frequency phase-frequency and phase-power coupling indicates that the frequency and power of the faster wave is modulated by the phase of the slower oscillation, respectively (Jensen and Colgin, 2007; Belluscio et al., 2012). For a more thorough review on the significance of each of these interactions as they relate to cognition see (Axmacher et al., 2006; Lisman and Buzsáki, 2008; Colgin, 2013; Lisman and Jensen, 2013).

Phase coherence is the relationship of two oscillations of the same frequency across different electrodes. For example, there can be phase-phase coupling of oscillations such that the phases of each ongoing oscillation are in sync (i.e., the peak of one oscillation always occurs in the same phase of a second oscillation measured at a second site). Likewise, two similar frequencies can correlate in their power, independent of the phase. Specifically, as the power of an oscillation increases at one recording site, a similar increase in power is observed at a second electrode. These types of specific interactions suggest that oscillations are not simply a local phenomenon but instead have a role in network activity.

In addition to phase coherence, oscillations can organize the firing of individual neurons by summing together subthreshold excitatory inputs or organizing the firing procession of assemblies of neurons (O’Keefe and Recce, 1993; Skaggs et al., 1996; Tukker et al., 2007). While there are different implications for the specific type of interaction observed, in general interaction between rhythms supports neural communication, plasticity, formation of functional ensembles and consolidation of long-term memories (Buzsáki and Draguhn, 2004; Fell and Axmacher, 2011; Belluscio et al., 2012). The common variable is that oscillatory patterns contribute to higher order information processing including the formation of neuronal ensembles.

Ensemble formation, or the linking of a group of cells, is at the heart of information processing (Fries, 2005; Buzsáki, 2010). Oscillations are capable of promoting ensembles partly through temporally precise segregation and boosting of communication between some groups of neurons. Theta-gamma phase modulation (cross frequency phase-amplitude coupling) can explain how one region of the hippocampus, say CA1, can be involved in multiple networks virtually simultaneously. For instance, CA3-CA1 shows the greatest coherence and phase locking of single unit firing in the slow gamma band (gammaS: 25–50 Hz), which peaks in the early descending phase of the CA1 theta. On the other hand, medial EC (MEC)-CA1 has high coherence at mid gamma frequency (gammaM 50–90 Hz) which is strongest at the peak of theta (Colgin et al., 2009; Belluscio et al., 2012; Schomburg et al., 2014). Thus, CA1 inputs are segregated across the phase of theta cycle and therefore individual pyramidal neurons have the potential to temporally align with multiple ensembles within a phase of theta (for detailed discussion, see Buzsáki and Schomburg, 2015). Alternating back and forth between functional networks could be important, as an example, for shifting between encoding new and retrieval of previous information (Montgomery and Buzsáki, 2007; Colgin et al., 2009), assembling discrete stimuli into a single representation (Gray et al., 1989; Engel et al., 1991), selective attention/gain modulation (Fries et al., 2001) and associative binding (Headley and Paré, 2013). Critically, in many cases theta provides the temporal structure for local gamma, while simultaneously coupling cell assemblies between regions and “chunking” (Buzsáki, 2010) information into discrete processing units (Senior et al., 2008; Buzsáki and Moser, 2013; Colgin, 2013). Given that theta is altered post-TBI (Fedor et al., 2010; Lee et al., 2013, 2015), it will be important for future studies to examine how TBI may affect these tightly coupled interactions and whether changes in these interactions might underlie injury-induced behavioral deficits.

Theta modulation can also strengthen synaptic connections and organize information flow. For instance, hippocampal theta phase locks local cortical gamma activity across multiple regions and the firing of individual cortical neurons (Sirota et al., 2003; Hyman et al., 2005; Jones and Wilson, 2005; Siapas et al., 2005; Fujisawa and Buzsáki, 2011). Convergence of cortical inputs onto the hippocampus coincides with a time (i.e., theta phase) that is optimized to support hippocampal synaptic plasticity (Berry and Thompson, 1978; Huerta and Lisman, 1995; Seager et al., 2002). In turn, local hippocampal plasticity is shaped by a difference in gamma phase-phase synchronization between subfields. During tonic REM sleep CA3-CA1 gamma coherence is decreased, while dentate gyrus-CA3 gamma is increased. This releases CA1 from CA3 recurrent collateral control and allows the dentate gyrus to modify CA3 synaptic activity. However, during brief interspersed periods of phasic REM theta and gamma coherence across all three subfields is increased and so is CA1 firing (Montgomery et al., 2008). Thus, it seems that CA1 is excluded until hippocampal information is transmitted back to the cortex (Buzsáki, 1989; Wilson and McNaughton, 1994; Ji and Wilson, 2007). Shifting between local cell assemblies ensures accurate transmission of information, discrete synaptic modifications free of interference and a receptive receiver to form an ensemble. Unfortunately, it is yet to be determined if these interactions are affected by TBI and if they are correlated with cognitive and behavioral deficits.

While to this point we have focused on theta, ensemble formation can be modulated at other frequencies. For example, during NREM sleep cortical slow oscillations (0.02–0.8 Hz) drive the cortex to alternate between a depolarized and a hyperpolarized (up/down) state (Steriade et al., 1993a; Cowan and Wilson, 1994; Timofeev et al., 2001). This slow oscillation also propagates to the thalamus and the hippocampus. A depolarized cortical state is associated with thalamacortical spindles which can bias high frequency CA1 burst activity, commonly referred to as sharp wave-ripples (Buzsáki et al., 1992; Battaglia et al., 2004; Mölle et al., 2006; Buzsáki and Silva, 2012). These ripples are synchronized to a particular phase of the spindles and drive the activation of specific cortical ensembles (Siapas and Wilson, 1998; Sirota et al., 2003; Isomura et al., 2006; Wierzynski et al., 2009). This hippocampo-cortical interplay may bind hippocampal output with coactive cortical ensembles. Together, these interactions have the added effect of associating two different networks in the spirit of coordinating information storage and promoting formation of long term memories through reciprocal excitation of ensembles. Ultimately, depending on the effect of TBI these ensembles could be as important of targets as those organized by theta oscillations.

It is important to note that the very functions ascribed to oscillations are perturbed in TBI patients. On a variety of neuropsychological exams patients score worse on tests of attention, concentration, working memory, reaction time, judgement and measures of effort (Rimel et al., 1981; Levin et al., 1988a; McDowell et al., 1997; Bales et al., 2009). These failures of information processing consolidate into deficiencies in verbal and visual memory, episodic memory, multitasking, executive function and cognition (Levin et al., 1988b; Hanks et al., 1999; Millis et al., 2001; Alvarez et al., 2003; Bales et al., 2009; Bootes and Chapparo, 2010; McCauley et al., 2014; Mäki-Marttunen et al., 2015). Deficits in higher order information processing are compounded by sleep-wake disturbances (Kempf et al., 2010; Shay et al., 2014; Skopin et al., 2015). TBI patients report a range of sleep-related disorders including difficulty falling asleep, more nighttime awakenings and daytime naps, increased fatigue, and prolonged sleep (Parcell et al., 2006; Kempf et al., 2010; Mathias and Alvaro, 2012). These sleep deficits are accompanied by altered REM and NREM sleep as detected by nighttime scalp recordings, as well as changes in nocturnal hormone secretion (Parsons et al., 1997; Frieboes et al., 1999). Given the overlap between functions associated with oscillations and observed deficits in TBI patients, additional research is critical to understand whether dysfunction can be ascribed to an alteration in oscillations. Moreover, if there is a relationship between oscillations and outcome in brain injured patients treatments aimed at modulating EEG are seemingly an appropriate starting point.

## Altered EEG After TBI

There are data that clearly indicate that TBI alters oscillations both in pre-clinical models as well as in patients (for detailed table on altered EEG, see Thatcher et al., 1989; Wallace et al., 2001; Rapp et al., 2015). In rodent models, there is a pronounced decrease in alpha, beta, delta and theta power following mechanical injury (Dixon et al., 1987; Ishige et al., 1987; McIntosh et al., 1987; Paterno et al., 2016). While most of these reductions return to baseline levels within minutes to hours after the injury (Sullivan et al., 1976; Dixon et al., 1988; McIntosh et al., 1989), some, like diminished theta, can persists for as long as 8–10 weeks after the insult (Fedor et al., 2010). A prolonged decrease in theta power is accompanied by other neurophysiological irregularities, even in brain regions spared from significant cell death. One such area, the CA1 subfield, exhibits altered excitation and inhibition, reduced LTP and pathological spine anatomy days to weeks after injury (Reeves et al., 1997; Sick et al., 1998; Sanders et al., 2000; Schwarzbach et al., 2006).

Analysis of human TBI patients resembles the reported prolonged recovery of EEG in experimental models of TBI. While modifications in brain activity can be seen as early as 24 h even after a subconcussive head trauma (Johnson et al., 2014), altered EEG following TBI can last for years after injury (Thatcher et al., 1989; Alvarez et al., 2008; Kempf et al., 2010; Slobounov et al., 2012). The changes in the EEG are not confined to a single oscillatory band, as they have been reported for the alpha, beta, delta, theta and gamma range (Alvarez et al., 2008; Tomkins et al., 2011; Rapp et al., 2015). Alterations of EEG activity are not state dependent, as changes are observed when a patient is at rest (Virji-Babul et al., 2014; Borich et al., 2015), actively moving (Slobounov et al., 2012) and during sleep (Parsons et al., 1997; Frieboes et al., 1999). In fact, abnormalities in scalp EEG are so consistent in patients they have been used to differentiate between injured and non-injured subjects, classify the severity of the injury, and some suggest, predict long term outcome after TBI (Thatcher et al., 1989, 1991, 2001; Alvarez et al., 2003; Arciniegas, 2011). For example, one of the criteria used to diagnose mild TBI many months after injury is an increase in coherence and a decrease in phase offset between frontal and temporal lobes, along with a decrease in power between frontal and posterior cortical regions (Thatcher et al., 1989). Furthermore, reversal of pathological EEG power ratio with administration of a neurotrophic peptide correlated with improvement in attention and working memory (Alvarez et al., 2008). The persistence of an abnormal EEG after a head injury suggests a potential link to prolonged psychological symptoms.

As is clear from the previous sections, it is not rigorous enough to determine that EEG is altered following injury. Unfortunately for the patient, it is also unlikely that there is a single mechanism that can easily explain why the EEG has changed. Therefore, it is critical to identify a starting point for research. While many neural systems and processes may be affected by a head injury, of particular interest (in our laboratory) is hippocampal dysfunction and the generation/maintenance of theta. TBI alters hippocampal neurotransmitter systems involved in the generation of theta, including acetylcholine, glutamate and GABA (Saija et al., 1988; Faden et al., 1989; Katayama et al., 1990; Robinson et al., 1990; Marshall et al., 2002). Rapid and prolonged increases in neurotransmitter levels act on local receptors causing long-lasting adaptation (Miller et al., 1990; Delahunty, 1992; Jiang et al., 1994; Delahunty et al., 1995; Schwarzbach et al., 2006). Thus, even after the injury-induced alteration of extracellular concentration of neurotransmitters returns to basal levels, modified receptors may have an ectopic response to subsequent activation, potentially affecting the timing and strength of receptor coupled processes essential to rhythm generation (Lyeth et al., 1992; Fineman et al., 1993; Kato et al., 2007; Marcoux et al., 2008). Another consequence of an intense glutamate discharge is excitotoxicity (Choi, 1988). This cell loss is readily observed in CA3 and dentate gyrus (Hicks et al., 1993; Floyd et al., 2002; Witgen et al., 2005), both of which are contributing nodes to CA1 theta (Bland, 1986; Kocsis et al., 1999; Marshall et al., 2002). Within the dentate gyrus, GABAergic interneurons in the hilus seem to be especially vulnerable to excitotoxicity, due to an increased excitatory drive onto their glutamatergic receptors (Tóth et al., 1997b; Hunt et al., 2011). Consequently, GABAergic cell death profoundly alters the excitability of not only the dentate gyrus, but the hippocampus as a whole leading to deficits in LTP and theta generation (Reeves et al., 1995, 1997; van den Pol et al., 1996; Witgen et al., 2005; Mtchedlishvili et al., 2010; Dinocourt et al., 2011). This hippocampal pathological process results in delayed degeneration in the MSN, a critical pacemaker for theta generation (Leonard et al., 1997). More specifically, cholinergic neurons within the septum show a marked susceptibility to cell death days to weeks following mild/moderate fluid percussion (Leonard et al., 1994; Schmidt and Grady, 1995) and a controlled cortical impact injury (Dixon et al., 1997). Such neuronal atrophy leads to enlarged ventricles and a proliferation of astrocytes, detected up to a year after TBI (Smith et al., 1997). Furthermore, downstream structures to the MSN, such as the hippocampus, show changes consistent with cholinergic hypofunction. In order to compensate for a decrease in evoked cholinergic neurotransmission (Dixon et al., 1996), there is an increase in the protein responsible for packing acetylcholine into presynaptic vesicles, downregulation of inhibitory autoreceptors and a hypersensitivity hippocampal cholinergic receptors and subsequent response of 2nd messengers (Jiang et al., 1994; Delahunty et al., 1995; Ciallella et al., 1998). MSN function could be further encumbered by post-traumatic epilepsy (Santhakumar et al., 2001; Frey, 2003; Pitkänen and McIntosh, 2006). Chronic seizure activity is related to a decrease (Garrido Sanabria et al., 2006) and altered firing of putative theta generating GABAergic cells in the septum (Colom et al., 2006). These observations have fueled the hypothesis that at least temporal lobe epilepsy in part arises due to an imbalance in septohippocampal theta and that theta stimulation could potentially be used as antiepileptic (Kitchigina et al., 2013; Fisher, 2015).

Degeneration and white matter damage also likely interfere with normal patterns of brain oscillations. Axonal abnormalities may arise from the initial shearing forces from the impact and an ionic imbalance in the extracellular space (Hicks et al., 1995; Graham et al., 2000; Li et al., 2014). Loss of ionic equilibrium leads to axonal increase of Ca^2+^ permeability, calpain activation, mitochondrial dysfunction and eventually breakdown of the cytoskeleton (Maxwell et al., 1997; Johnson et al., 2013). These changes may culminate in deafferentation/denervation and inappropriate synaptic plasticity (Povlishock and Katz, 2005; Hunt et al., 2011). Many of these axonal changes can be detected weeks after the insult and correlate with behavioral abnormalities (Kempf et al., 2010; Spain et al., 2010). It is not surprising then that compromised axonal structure and function results in irregular oscillatory interactions, even years after the injury. These structural deformities along with neurochemical aberrations contribute significantly to the observed deficits in the propagation of functionally relevant hippocampal theta (Fedor et al., 2010), and subsequently brain function after TBI (Hanks et al., 1999; Millis et al., 2001). These data clearly indicate that multiple TBI-induced mechanisms can play a role in altered brain oscillations and their interactions thus contributing to long-term impaired cognition.

## Theta DBS

Pathologies associated with TBI are wide-ranging, occurring at the molecular, physiological and structural level. These alterations in turn may lead to changes in network activity, affecting neural communication and plasticity. Abnormal rhythm generation could potentially hinder and prolong recovery after a TBI insult (Thatcher et al., 1991; Tomkins et al., 2011). Furthermore, once a patient has progressed out of the acute post-injury phase of the disease, neuroprotection is no longer a viable therapeutic option. Therefore, there is an urgency to develop treatment strategies for TBI patients who have chronic disability. DBS represents a potential intervention that can drive neural networks, improve neurophysiology and ultimately behavioral outcome in a subset of brain-injured patients. The advantage of neurostimulation, say over pharmacology, is its ability to target specific regions, inherent higher temporal resolution and ability to generate specific patterns of electrical input, all of which are critical factors in the generation and interaction of oscillations. Furthermore, neurostimulation has shown promise in alleviating symptoms in motor, cognitive, behavioral and psychiatric conditions (Brunoni et al., 2011; Lozano and Lipsman, 2013; Suthana and Fried, 2014). The relative success of DBS in each specific situation is determined by a growing body of parameters, beyond the scope of this article to survey (Kuncel and Grill, 2004; Butson and McIntyre, 2007; Birdno and Grill, 2008). Therefore, given the focus of this review we will highlight the potential use of one stimulation strategy, the use of low frequency stimulation within the theta range.

Exogenous induction of theta in structures like the hippocampus can improve cognitive processes in experimental animals. Hippocampal theta can be achieved either with direct hippocampal stimulation or by targeting afferent structures such as the fornix or MSN. Using rodent models, pre-training stimulation of MSN decreased the time to acquire discriminatory learning (Deupree et al., 1982) while post-training stimulation facilitated memory consolidation (Landfield, 1977; Wetzel et al., 1977). These results mirror the positive correlations observed between endogenous theta and enhanced acquisition and retention (Landfield et al., 1972; Berry and Thompson, 1978; Seager et al., 2002; Mandile et al., 2003; Mitchell et al., 2008). Importantly, the uniqueness of these findings lies not in the MSN *per se*, but in the theta oscillation. The critical role of theta oscillations specifically was revealed in studies were theta stimulation of the fornix was able to drive hippocampal theta oscillations following chemical inactivation of the MSN (Green and Arduini, 1954; Petsche et al., 1962; Vertes et al., 2004). Not only did stimulation drive theta, but also rescued the behavioral impairment (McNaughton et al., 2006). Further substantiating the selectivity of theta range, high frequency MSN stimulation does not facilitate mnemonic processes (Landfield, 1977; Wetzel et al., 1977). Therefore hippocampal theta, generated endogenously or extrinsically, plays a critical role in neural computations supporting animal cognition.

The beneficial effects of stimulation in the theta range are not limited to cognitive processes. For example, low frequency stimulation has been shown to be beneficial after an acute spinal cord contusion where an 8 Hz stimulation of the raphe nucleus improved motor coordination and sensory processing, increased white matter integrity and reduced astrocytosis (Hentall and Burns, 2009; Hentall and Gonzalez, 2012). Therefore it is possible that stimulation of theta following TBI might also have indirect effects that could improve the hippocampal milieu post-injury facilitating anatomical as well as physiological recovery. Epilepsy treatment is also closely associated with low frequency stimulation. Effective reduction of kindling from a 60 Hz induced seizure is achieved with 3–5 Hz stimulation (Gaito et al., 1980; Kile et al., 2010; Koubeissi et al., 2013). Building on this framework, Fisher recently proposed a novel hypothesis that MSN stimulation in the theta range may benefit patients with epilepsy (Fisher, 2015). Therefore stimulation following severe TBI might also have the added benefit of reducing or preventing post-traumatic epilepsy.

## Theta DBS in TBI Models

**Figure 4:**
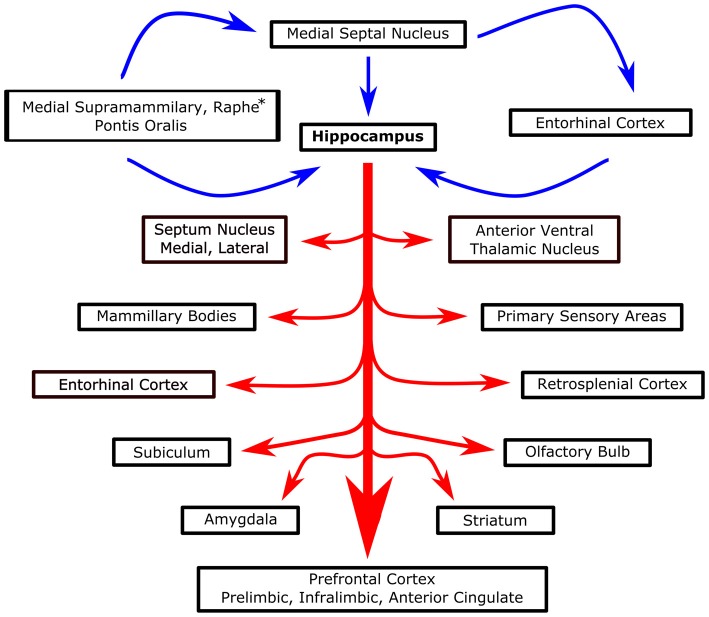
**Systems level overview of septohippocampal theta.** Blue arrows represent proposed theta generators and essential modulators of hippocampal theta. Red arrows, stemming from the hippocampus, represent various structures that are known to be modulated/interact with hippocampal theta. *Raphe only projects to the hippocampus, not MSN.

Recently, several studies have described the restorative effect of theta stimulation after TBI injury. Lee et al. (2013, 2015) stimulated the MSN at 7.7 Hz and recorded an increase in hippocampal theta power along with better spatial performance in the Barnes maze following a moderate lateral fluid percussion injury (Figure 4). Several stimulation controls bolstered the hypothesis that theta band stimulation was specifically augmenting the septohippocampal system in TBI rats. In particular, successful MSN stimulation in the theta range was intensity specific, there was no effect on overall motor output (i.e., distance traveled) and MSN stimulation at 100 Hz did not rescue the deficit in spatial performance (i.e., spatial search strategy) in the maze (Lee et al., 2015). Moreover, the authors concluded the effect was restorative and not simply enhancing function as similarly stimulated sham animals experienced no improvement in spatial learning. Likewise, the Hentall group observed positive effects on spatial memory in the watermaze and forelimb reaching movements when, following lateral fluid percussion, they stimulated the raphe nucleus, part of the ascending system that generates theta, at 8 Hz (Vertes et al., 2004; Carballosa Gonzalez et al., 2013). In conjunction with behavioral outcomes, stimulation reversed cortical cell loss, white matter degeneration and decreases in cortical and hippocampal levels of cAMP, an intracellular second messenger (Carballosa Gonzalez et al., 2013). These proof of principle studies illustrate the potential of theta stimulation to augment physiological and behavioral outcome following TBI.

Importantly, theta stimulation has not been the only successful stimulation paradigm observed in experimental models of TBI. In a model of mild TBI, theta burst stimulation (TBS) was able to rescue working memory in the T-maze delayed non-match to sample task. Rather than using a continuous single pulse (7.7 or 8 Hz) fixed stimulation (Sweet et al., 2014) used a TBS protocol to stimulate the fornix, specifically with five 50 ms long bursts of high frequency (200 Hz) pulses per second. The hypothetical advantage of TBS as compared to continuous theta stimulation is that the 200 Hz gamma stimulation partially recapitulates endogenous patterned firing at a physiologically relevant theta frequency. In fact TBS has been demonstrated to induce long lasting LTP (Rose and Dunwiddie, 1986; Staubli and Lynch, 1987; Diamond et al., 1988; Kirkwood et al., 1993). Accordingly, Sweet et al. (2014) reported that TBS, but not low (5 Hz) or high (130 Hz) frequency stimulation of the fornix, improved performance of TBI rats in the T-maze. Spatial memory in the water maze was also improved with TBS; however, 5 Hz stimulation was not tested.

There are several key takeaways from these two successful stimulation paradigms. The first is that task might matter. There is evidence that, depending on task, there is a shift in the frequency of the theta oscillation (Kramis et al., 1975; Watrous et al., 2013). Therefore, the specific frequency within the theta range may be critical to improving outcome and the target may be different for different behaviors. Following that reasoning, while 5 Hz stimulation may not have improved T-maze performance, it is possible that 7.7 (or some other frequency) may have. In fact, in a study by McNaughton et al. (2006), it was observed that to optimally restore behavior in the watermaze after chemical inactivation of the MSN, it was best to stimulate the fornix with an endogenous EEG pattern recorded from the supramamillary nucleus as compared to fixed 7.7 Hz or an irregular theta stimulation pattern (with an average frequency of 7.7 Hz). These data indicate that not all “theta” is the same, and that the specific frequency within the theta band may be highly relevant.

The fact that these initial reports of DBS in TBI did not report completely homogenous results is worth noting. The data highlights the need for additional research to investigate the large parametric space available for potential stimulation parameters. There are many additional variables to be considered: intermittent vs. constant stimulation; endogenous stimulation (where theta is recorded from an uninjured site and played back in the injured hippocampus as in McNaughton et al., 2006) vs. exogenous fixed frequency; theta burst vs. single pulse; variants in voltage, pulse width and square as compared to sinusoidal; which regions, nuclei or subfields to target; at which point during the task/behavior to stimulate or whether to stimulate offline during sleep or to stimulate relative to an endogenous oscillation independent of the behavior. A closed loop system where stimulation was based on the recorded EEG (from a different region) would subsequently be amenable to biofeedback (Wallace et al., 2001; Rosin et al., 2011; de Hemptinne et al., 2015).

Different stimulation parameters will not only influence the efficacy of the treatment, but also most likely the extent of unintended effects. The most commonly reported adverse events are related to the implant rather than stimulation and include inflammation, headache, pain at the implant site, and mild paresthesia surrounding the implant (Kenney et al., 2007; Fisher et al., 2010; Salanova et al., 2015). However, in studies of stimulation for treatment of epilepsy there are reports of cognitive dysfunction, depression and suicide in a small number of patients (Bergey et al., 2015; Salanova et al., 2015). Therefore it will be important to monitor which symptoms TBI patients report receiving low frequency stimulation of the septohippocampal system. While complications with the surgery and device itself are minimal, as argued by Fisher (2015) in his proposal to stimulate the MSN in epileptic patients, there are however potential risks of eliciting seizures or promoting addiction to constant stimulation (for discussion on long-term safety of DBS, see Kenney et al., 2007). These potential risks (e.g., prior epileptic activity, predisposition to addiction, mood/affect disorders) should be taken into consideration when enrolling patients so as to minimize potential harm. The inclusion criterion could be further refined based on the mechanism of action of neurostimulation in the theta range. If DBS is masking an enduring effect or if it is restorative will potentially influence the type of therapy one gets, such as; when should DBS be administered relative to the injury and in response to what type of injury? Will immediate intervention interfere with the healing process or will waiting too long make the system unamenable? How long will the benefits of DBS persist, if stimulation is discontinued? Should the treatment continue indefinitely or should there be a clinical marker/threshold to stop or augment the stimulation? Thus, there is a clear need for considerable pre-clinical animal work and potentially computational modeling to better understand and explore the complex parameter space that is DBS and the mechanisms behind it, if we are to optimize the potential of neurostimulation for clinical translation.

## Conclusion

After years of research, there are few proven interventions that reduce injury-induced cellular cascades and ultimately, cell death and dysfunction following TBI. While the latest census estimates over 5.3 million patients live with chronic disability, it is clear that that number has grown and continues to grow. Therefore, there is a clear need for pre-clinical research expressly focused on the injured nervous system in the chronic stages of the disease. Oscillations are known to play a key role in physiological circuit function, whether it is the progression of oscillations through the sleep cycle or theta oscillations in the hippocampus. Initial evidence suggests that injury-induced disruption of these oscillations has a profound impact on neural connectivity and behavior. In fact, changes in EEG can be used as a biomarker to confirm mild and moderate TBI. Additionally, limited studies of DBS in brain injured rats demonstrate that the injured brain can be modulated by entraining or replacing oscillations, with improved outcomes. Future preclinical studies are needed to explore a very large parametric space that spans not only multiple stimulation targets and paradigms but also different injury mechanisms as well as a range of cognitive behavioral tasks and dependent measures, extending beyond spatial navigation. The potential for DBS is clear. We believe that further research into electrical neuromodulation of the injured brain will result in an exciting avenue to promote behavioral, cognitive and neurophysiological recovery following TBI.

## Author Contributions

AP, KS, and GGG contributed to the conception, writing and editing of the manuscript. AI, DJL contributed significantly to the conception and editing of the manuscript.

## Conflict of Interest Statement

The authors declare that the research was conducted in the absence of any commercial or financial relationships that could be construed as a potential conflict of interest.
